# Optimizing Lifestyle Before and After Surgery: A Qualitative Stakeholder Analysis Among Healthcare Professionals

**DOI:** 10.1177/15598276251355917

**Published:** 2025-06-29

**Authors:** Melissa J. J. Voorn, Evy E. J. Jetten, Celine Franssen, Frederik O. Lambers Heerspink, Joop L. M. Konsten, Esther R. C. Janssen, Maryska L. G. Janssen-Heijnen

**Affiliations:** 1 Department of Rehabilitation Medicine, Care and Public Health Research Institute, 5211 Maastricht University, Maastricht, The Netherlands (MJJV); 2 Department of Epidemiology, GROW Research Institute for Oncology and Reproduction, Faculty of Health, Medicine and Life Sciences, 5211 Maastricht University, Maastricht, The Netherlands (MJJV, MLGJ); 3 Centre of Expertise in Rehabilitation and Audiology, Adelante, Hoensbroek, The Netherlands (MJJV); 4 Department of Orthopaedic Surgery, 8187 VieCuri Medical Centre, Venlo, The Netherlands (EEJJ, FOLH, ERCJ); 5 Department of Clinical Epidemiology, 8187 VieCuri Medical Centre, Venlo, The Netherlands (EEJJ, MLGJ); 6 Department of Orthopedic Surgery, Care and Public Health Research Institute, 5211 Maastricht University, Maastricht, The Netherlands (EEJJ, FOLH); 7 Practice for Physical Therapy, Fysio Centrum Tegelen, Tegelen, The Netherlands (CF); 8 Department of Surgery, 8187 VieCuri Medical Centre, Venlo, The Netherlands (JLMK); 9 IQ Health Science Department, Radboudumc, Nijmegen, The Netherlands (ERCJ); 10 School of Allied Health, HAN University of Applied Sciences, Nijmegen, The Netherlands (ERCJ)

**Keywords:** lifestyle interventions, social support, surgical care, perioperative care, interdisciplinary collaboration

## Abstract

Healthcare professionals play a crucial role in promoting lifestyle changes among patients with cancer or osteoarthritis by identifying risk factors and motivating engagement in lifestyle interventions. Prehabilitation, defined as the process of optimizing patients’ physical and mental health before surgery, is increasingly recognized as an essential component of perioperative care. This study explores healthcare professionals’ perspectives on implementing lifestyle interventions as part of prehabilitation programs in surgical care. This study aims to investigate the experiences, preferences, and perceptions of healthcare professionals regarding (1) the content of lifestyle interventions to support healthy behaviors and (2) the optimal organizational and social context for delivering these interventions. A qualitative study was conducted using semi-structured interviews with medical specialists, nurses, dietitians, physiotherapists, and psychologists. The COM-B model guided the exploration of capability, opportunity, and motivation for behavior change. Thematic analysis identified key themes. Healthcare professionals (n = 25) emphasized the need for tailored lifestyle advice and social support integration. Barriers included limited time, insufficient training in communication techniques, and organizational constraints. Enablers of patient engagement were effective communication and involving loved ones. Multidisciplinary collaboration, centralized referral systems, and a lifestyle front office were proposed as solutions. Healthcare professionals emphasized personalized approaches to address socioeconomic and literacy barriers. To promote lifestyle interventions, healthcare professionals need communication training, interdisciplinary collaboration, and structured support systems. Addressing organizational constraints and integrating personalized, patient-centered approaches are essential to overcoming socioeconomic barriers and ensuring long-term adherence to a healthy lifestyle.

“Healthcare professionals identified systemic barriers for implementing lifestyle interventions, including insufficient time, resource limitations, and lack of financial support.”

## Introduction

Initiating and maintaining a healthy lifestyle is vital for individuals undergoing significant medical treatments, such as major surgery. Major surgery typically refers to procedures involving substantial physiological stress, general or regional anesthesia, and a prolonged recovery period. Many of these patients are vulnerable, with limited physiological and mental reserves.^1-3^ This increases their risk of complications and may delay their recovery process.^4,5^ A focus on healthy lifestyle changes throughout the perioperative journey helps patients regain independence and achieve better overall well-being.^6,7^ A healthy lifestyle has been shown to contribute to better clinical outcomes, including fewer complications, faster recovery, improved quality of life and social participation.^5,8-10^

The World Health Organization emphasizes that a healthy lifestyle involves a balance of informed health choices, including proper nutrition, regular physical activity, emotional and spiritual wellness, adequate sleep, avoidance of harmful substances, stress, and unhealthy work-life balances.^11^ Prehabilitation, which includes targeted lifestyle interventions, has proven effective especially in frail patients.^6,7,12-15^ Despite the benefits of prehabilitation, adherence to prehabilitation programs remains a challenge, with adherence rates reported between 50% and 75%.^15-21^ Guidance and a supportive environment can improve adherence to prehabilitation and rehabilitation programs and help to maintain a healthy lifestyle during the perioperative phase.

To create a lasting impact on patients’ lifestyles, it is essential to understand the factors that drive motivation and behavioral change in the context of initiating and maintaining a healthy lifestyle, during the perioperative period.^17^ While integrating lifestyle interventions into patients’ daily routines is crucial, it is equally important to address the barriers that healthcare professionals face in discussing lifestyle-related topics during consultations. Evidence suggests that healthcare professionals may struggle with these conversations due to a perceived lack of time, limited training in lifestyle counseling, uncertainty about the effectiveness of interventions, or concerns about patient receptivity.^22^ Understanding these challenges can help in designing tailored strategies to support professionals in integrating lifestyle conversations into routine care.^22^ Key strategies for healthcare professionals to improve adherence to healthy behaviors include ensuring practicality, enhancing motivation, and optimizing social support. For example, guidance from a trained lifestyle professional may help patients stay focused and motivated.^23,24^ A comparative study has highlighted significant differences between Swedish and American healthcare professionals regarding their engagement in lifestyle counseling, the importance they attribute to it, and their perceived expertise in the area.^25^.^25^ This finding underscores the role of cultural and systemic factors in shaping healthcare professionals’ approach to lifestyle interventions. The way in which lifestyle interventions are integrated into healthcare settings plays a crucial role in adherence. Professional, social, and community-based support has been shown to enhance self-efficacy, motivation, coping capacity, and psychological well-being, all of which positively influence adherence.^24,26,27^ However, the extent to which healthcare professionals facilitate referrals to lifestyle support and their perceptions of effective intervention strategies can vary significantly across settings and medical fields. While existing studies offer general recommendations for optimizing the delivery of lifestyle interventions,^26,28-31^ there is limited insight into how these should be tailored for patients preparing for major surgery.

This study focuses on the perspective of healthcare professionals, who play a critical role in supporting patients in making lifestyle changes surrounding major surgery. The aim of this study was to explore the experiences and preferences of healthcare professionals involved in the care of cancer or osteoarthritis regarding^1^ the content of lifestyle interventions to support healthy behaviors and^2^ the optimal organizational and social context for delivering these interventions.

## Methods

### Study Design

This qualitative study utilized individual semi-structured interviews with healthcare professionals involved in the care of patients undergoing major surgery for cancer or osteoarthritis. Ethical approval for this study was obtained from the Medical Ethics Review Committee of azM/University Maastricht (reference number: 2023-0381).

### Study Population

Between January 2024 and July 2024, healthcare professionals involved in the treatment of patients with cancer or osteoarthritis at VieCuri Medical Centre were recruited. Participants included medical specialists, rehabilitation physicians, nurses, psychologists, dietitians, and physiotherapists; some of the included participants had with experience in prehabilitation for patients with cancer or osteoarthritis. No exclusion criteria were applied. Healthcare professionals were recruited via convenience sampling. Participants were identified based on the researchers’ knowledge of their involvement in the care of patients with cancer or osteoarthritis and/or their experience with prehabilitation. Identified professionals were contacted directly by email and invited to participate in the study. Upon receiving email consent, the researcher scheduled interviews with each participant. Written informed consent from healthcare professionals was obtained at the start of the interview by the researcher after discussion of the purpose and requirements of the study.

### Data Collection

Data was collected through one-on-one semi-structured interviews. Interviews were conducted in person at VieCuri Medical Centre or online (using Webex). One researcher (CF) conducted the interviews with healthcare professionals involved in cancer treatment, and one researcher (EJE) conducted the interviews with healthcare professionals involved in osteoarthritis treatment. A purposive sampling strategy was used to include a diverse group of healthcare professionals involved in surgical prehabilitation. Recruitment continued until data saturation was reached, defined as the point when no new themes emerged in consecutive interviews.^25^

### Content of Interviews

A semi-structured interview guide was developed using an existing behavioral model. The Capability, Opportunity, and Motivation for Behavior (COM-B) model^32^ served as a framework for categorizing questions, which were designed to be open-ended (see Table 1). The COM-B model posits that behavior is influenced by three components: capability (e.g., physical skills and knowledge), opportunity (e.g., environment and social norms), and motivation (e.g., habits and beliefs). This model encompasses all factors that affect behavioral change.^32^ A single pilot interview with a physiotherapist was conducted to refine the interview guide based on their feedback.

**Table 1.** Interview Guide Based on the COM-B Model.

Capacity	
**1**	What is your definition of a healthy lifestyle?
**2**	How do you think a healthy lifestyle affects surgical outcomes?
**3**	Is it easy or difficult for you to assess whether someone needs social support (e.g., loved ones/caregivers, companions, peers, or experienced mentors) before surgery? What could help with this (e.g., screening/questionnaire)?
Opportunity	
**4**	Is there sufficient opportunity to discuss lifestyle in the consultation room? If not, how could this be best implemented? What prerequisites are needed (e.g., conversation cards, time, or a screening questionnaire)?
**5**	For which patients do you think lifestyle changes are most valuable? Which aspects of lifestyle are most important for your patients? Where should lifestyle interventions ideally take place?
**6**	What barriers exist for referring patients to lifestyle interventions, and what could make it easier to refer them?
**7**	Who do you think should be responsible for guiding lifestyle interventions?
Motivation	
**8**	How do you think patients maintain lifestyle changes before and after surgery? How can this be improved?
**9**	How do you involve patients’ relatives in providing lifestyle advice?
**10**	What role does social support play in initiating and maintaining a healthy lifestyle (e.g., from loved ones/caregivers, companions, peers, or experienced mentors)?
Behavior	
**11**	How do you address lifestyle with patients? What facilitates these discussions? What are barriers to discussing lifestyle?
**12**	How should patients ideally be referred for lifestyle interventions? When do you refer patients, and for what and to whom?
**13**	What role do healthcare professionals play in making social support a topic of discussion? Which healthcare professionals should address this, and when should it be discussed?
Other questions	
**14**	What are your thoughts on extending the time before surgery to allow for more lifestyle changes? How long do you think surgery could be postponed?
**15**	If a lifestyle support center (a place where patients can get help with lifestyle-related questions) were to be set up, what would this ideally look like?
**16**	Do you envision this in primary, secondary, or tertiary care?
**17**	If within the hospital, where should it be located?
**18**	How should accessibility be organized (e.g., opening hours, walk-in or by appointment)?

The interview questions focused on healthcare professionals’ perceptions of a healthy lifestyle, its impact on surgical outcomes, and their capability, opportunity, and motivation to assess patients' (social) support needs. Topics included barriers to lifestyle discussions, strategies to involve loved ones, and tools to aid these processes. Practical aspects such as time, resources, and referral pathways for lifestyle interventions were explored. Healthcare professionals were asked to reflect on their current practices and what future lifestyle support should look like in the form of a lifestyle front office.

### Data Analysis

The research team was multidisciplinary, including experts in rehabilitation medicine, behavioral sciences, and public health, all with prior experience in conducting qualitative research. Researchers had no established relationships with participants prior to the interviews. To ensure reflexivity, the team engaged in regular discussions to reflect on potential biases and their influence on data collection and analysis. The study design and reporting were informed by the COREQ (Consolidated Criteria for Reporting Qualitative Research) checklist to enhance transparency and methodological rigor. Data were analyzed according to thematic analysis principles and the standards for reporting qualitative research checklist.^33^ The interviews were recorded and transcribed verbatim. Transcripts were analyzed using inductive content analysis in ATLAS.ti version 9.^34^ The open codes were divided into subthemes and themes using thematic analysis.^34^ The first three interviews of each study population were independently fragmented, coded, and thermalized by two researchers (EJE and CF). Themes were discussed until consensus was reached by three researchers (EJE, CF, and MV). These themes were used as a base for coding the other transcripts.

## Results

### Recruitment and Sampling

A total of 25 interviews were conducted with healthcare professionals. Two surgical oncologists, two oncologist, two orthopedic surgeons, one urologist, two rehabilitation physicians, two sports physicians, four physiotherapists, two psychologists, two dietitians, three clinical nurse specialists, and three nurses participated. The mean age of the participants was 45 years (range: 24-62), with an average of 14 years of work experience. All participant characteristics are summarized in Table 2.

**Table 2.** Characteristics of the Healthcare Professionals.

Parameters		Population (n = 25)
**Sex** n (%)		
Female		14 (56)
Male		11 (44)
**Mean age in years (range)**		45 (24-62)
**Time working in the hospital in years (range)**		14 (2-35)
**Interview duration in minutes (range)**		36 (22-62)
**Profession** n (%)		
Medical specialists	Surgical oncologist	2 (8)
	Oncologist	2 (8)
	Orthopedic surgeon	2 (8)
	Urologist	1 (4)
	Rehabilitation physician	2 (8)
	Sports physician	2 (8)
Therapists	Physical therapist	4 (16)
	Psychologist	2 (8)
	Dietitian	2 (8)
Nurse	Clinical nurse specialist	3 (12)
	Nurse	3 (12)

### Themes

The analysis of the interviews identified five themes: competence of healthcare professionals, capability of patients, motivation of patients, collaboration, and organizational factors.

In the next paragraphs results are presented according to these themes. Final themes and subthemes were described and summarized on a code tree (Table 3). Collaboration refers to the current interactions, communication, and partnerships among healthcare professionals within and outside the hospital that facilitate lifestyle interventions. Organizational factors relate to the broader structural, systemic, and resource-related aspects that influence how lifestyle care is organized, supported, and sustained at an institutional or policy level.

**Table 3.** Code Tree.

**Open Codes**	**Subthemes**	**Themes**
Diet Exercise pattern Smoking-cessation Limited alcohol use Prehabilitation Limited time Lack of educational tools Insufficient training Lack of support	Knowledge healthy lifestyle	Competence of healthcare professionals
Discuss lifestyle with patient Tool to start conversation about lifestyle Discussing social support Involving loved ones in giving lifestyle advice Repetition is important Information sometimes limited to hospital stay Managing expectations	Communication skills	
Knowledge of the patient Low literacy Low educated Comorbidity Illness beliefs patient	Health literacy	Capability of patients
Involvement of relatives Involvement of environment Fellow sufferer contact Buddy system Experience expert	Social support	
Migration background Cultural differences Lack of financial resources	Social and community aspects	
Patients must be open to lifestyle changes Coping Self-discipline Awareness seems less in older patients Responsibility	Internal motivation patient	Motivation of patients
Influence from loved ones Influence from environment Surgery motivates patient to make changes Recovery of illness leads to relapse in old lifestyle pattern	External motivation patient	
Knowledge about referral External referral Internal referral Tool to support referral	Referral	Collaboration
Collaboration in the hospital Collaboration outside the hospital Monitoring role physician Not the role of all healthcare professionals	Division of roles between healthcare professionals	
Accessible contact Shared decision-making Healthcare professionals guiding role Patient has final responsibility	Healthcare professionals -patient relationship	
Collaboration with the municipality Support from the government Support from health insurers Lifestyle front office The right healthcare in the right place	Societal aspects	Organizational factors
Involvement perioperative care Intensive guidance during prehabilitation	Contact moments	

### Competence of Healthcare Professionals

Healthcare professionals highlighted that the importance of discussing lifestyle with their patients and providing tailored advice are not merely additional recommendations, but essential components of the recovery process, playing a key role in improving pre- and postoperative outcomes. They expressed the attitude that lifestyle conversations are fundamental, yet shared experiences of limited time, lack of educational tools, and insufficient training in discussing a healthy lifestyle were commonly cited as significant barriers to effective implementation. Many professionals reported feeling inadequately equipped to address the broad spectrum of lifestyle factors outside their specific areas of specialization.

“I refer patients for dietary advice or physical activity, but I often feel limited in my knowledge about other lifestyle factors, like stress or sleep.” - Medical specialist

Establishing trust with patients and communicating tactfully about sensitive topics, such as obesity, were viewed as critical skills. Some professionals expressed a preference for using tools like the “lifestyle wheel” to structure discussions and empower patients to take control of their health. They highlighted the need for more structured guidelines, digital tools, professional training in behavioral change techniques, and access to multidisciplinary teams and referral pathways to enhance the effectiveness of lifestyle interventions.

“Motivational interviewing helps us make patients think for themselves. It’s about asking the right questions, not telling them what to do.” - Therapist

Healthcare professionals recognized the value of prehabilitation programs, as these prepare patients for surgery through physical activity and provide them with insight into the role of the patient in the rehabilitation process. They shared experiences that repetition and clarity in communication reinforce lifestyle advice. Medical specialists expressed the attitude that they bear primary responsibility for lifestyle discussions but often face time constraints, prioritizing smoking-cessation, and leaving other lifestyle aspects to different disciplines.

The involvement of loved ones was frequently mentioned as important by healthcare professionals, as it often enhances the patient’s ability to sustain behavioral changes. However, challenges were also highlighted, such as the inability to directly refer partners of patients who smoke to smoking-cessation programs, which some professionals experienced as a barrier to collective behavioral change. Nurses stressed the role of social support in maintaining behavioral changes, but faced challenges in systematically assessing it due to the absence of standardized screening tools.

“I explain, if you want to work on a lifestyle factor, like quitting smoking, but the partner keeps smoking, then it won't work. So, you involve the partner and try to motivate them to join—not just for the patient, but also for themselves. I always explain that you’re not doing it for your partner but ultimately for yourself and your children or grandchildren.” - Medical specialist

### Capability of Patients

Healthcare professionals emphasized that patients’ physical and social conditions greatly influence their capability to implement lifestyle changes. For patients with cancer, lifestyle discussions were often framed as a necessity to improve treatment tolerance, though professionals experienced variability in patients’ motivation, particularly in later stages of illness. In contrast, patients with osteoarthritis were considered more suitable for lifestyle interventions, as physical activity directly impacted their pain and mobility, with benefits that were more immediate and tangible. The influence of socioeconomic status was also frequently reported, with lower-income patients encountering more obstacles, including limited resources, reduced access to care, and lower health literacy.

“Patients with a lower socioeconomic status often lack the resources or autonomy to make meaningful lifestyle changes. It’s not that they don’t want to, but they don’t know how to begin.” - Therapist

Many healthcare professionals observed that patients with multiple challenges, such as restricted mobility or high levels of stress, found it difficult to prioritize lifestyle improvements. For patients in critical conditions or experiencing significant pain, the emotional toll often outweighed their ability to focus on their lifestyle. Low health literacy and lack of health awareness were commonly reported barriers.

“There are entire groups who have never received adequate education (low-literacy populations)…they don’t understand the importance and can’t comprehend it. We can talk all we want, but they just don’t see it.” - Nurse

Healthcare professionals noted that some patients with a diagnosis of cancer perceived an impending operation as a wake-up call, motivating them to improve their lifestyle, while others, particularly those facing end-of-life care, showed little interest or energy for such changes. They emphasized the need for personalized approaches, taking into account individual circumstances and coping styles.

*“There’s no point in referring patients who say upfront they won’t follow through. You need highly motivated patients. Otherwise, it’s a waste of resources for those who are not motivated.” - Medical specialist*

### Motivation of Patients

The motivation of patients to initiate lifestyle changes was described by healthcare professionals as a pivotal factor in initiating and maintaining health improvements. Healthcare professionals observed that patients were often motivated by the immediate necessity of surgery, as they recognized the benefits of a healthy lifestyle for their recovery. This motivation often diminished according to healthcare professionals once the acute necessity had passed. Healthcare professionals indicated that professional and social support, and clear communication of expectations are essential for maintaining long-term motivation.

“When patients realize that surgery is coming, they’re often more motivated to make changes. But once the surgery is over, that motivation tends to drop.” - Medical specialist

By the healthcare professionals, social support was emerged as an enabler of motivation, with loved ones, friends, and caregivers often playing a vital role in encouraging patients. For individuals with limited social networks, the lack of support was seen as a major barrier to lifestyle changes, increasing the risk of isolation and inactivity. Peer support groups, buddies, and online resources were occasionally suggested as additional avenues for support, though some healthcare professionals expressed concerns about misinformation from unregulated sources.

“People with limited social connections are more likely to stay inactive and isolated, leading to a less active lifestyle.” - Nurse

“Peer groups might help, but I don’t use them much. Patients who search online for peer support often end up panicking over the conflicting information they find.” - Medical specialist

Financial constraints, cultural differences, and migration backgrounds were also identified as barriers for motivation. Healthcare professionals suggested municipal support and community-based initiatives as potential solutions for patients facing financial or cultural challenges.

### Collaboration

Healthcare professionals emphasized the importance of interdisciplinary collaboration in supporting lifestyle interventions. Multidisciplinary team meetings were cited as effective mechanisms for coordinating care, though some healthcare professionals noted that these meetings could be utilized more strategically to address lifestyle issues. The lack of centralized coordination for lifestyle interventions was a recurring theme, with healthcare professionals expressing a need for a more structured approach to integrate various disciplines, such as dietitians, physiotherapists, and psychologists, into a coordinated care strategy.

“It’s essential to work with other disciplines, but we often miss opportunities to align care because there’s no central coordination.” - Therapist

The general practitioner was often regarded by healthcare professionals as a central figure in referrals to lifestyle interventions. The fragmented nature of collaboration between general practitioners and other specialists was identified by healthcare professionals as a barrier. Several healthcare professionals advocated for the establishment of a centralized “lifestyle front office” to streamline referrals and provide patients with access to a wide range of support services tailored to their needs.

“I think we all feel responsible for our part. It would be great if everything came together. Right now, everyone does their own thing. Ultimately, I think the general practitioner remains the primary contact.” - Therapist

“The general practitioner is supposed to be the central figure, but they’re already overloaded. We need a lifestyle front office to take over some of the coordination.” - Medical specialist

Healthcare professionals consistently expressed that interventions should be patient-centered and delivered in settings that align with the patient’s daily life eventually by the appropriate disciplines in either secondary or primary care. Some healthcare professionals viewed lifestyle interventions as a natural fit within primary care, while others indicated that they were better suited for specialized secondary care. Some of healthcare professionals referred patients to lifestyle interventions through a digital platform, while others made limited use of external referrals. Most professionals acknowledged that they themselves had an important role to play in supporting lifestyle changes, either directly or through collaboration with other disciplines.

### Organizational Factors

Healthcare professionals consistently highlighted organizational barriers as impediments for implementing lifestyle counseling effectively. They pointed out the lack of structural support within their organization, such as lifestyle clinics that focus on promoting healthy living and managing lifestyle-related diseases, or integrated systems for providing comprehensive guidance. According to the healthcare professionals, comprehensive guidance involves not only providing information but also offering practical support in areas like physical activity, nutrition, stress management, and mental well-being, helping patients navigate various aspects of initiating of maintaining their healthy lifestyle. Time pressures and resource limitations were frequently mentioned by healthcare professionals, who explained that they were often forced to prioritize patients with immediate, acute health needs over long-term lifestyle support, such as chronic disease management or preventive care programs.

“We need funding for lifestyle discussions—time spent on this should be billable, so clinics can structure their schedules accordingly. So, direct patients to a lifestyle front office for more comprehensive support.” - Medical specialist

Healthcare professionals stressed the need for substantial investments in preventive care, such as combined lifestyle interventions and long-term coaching programs. They mentioned that digital tools were sporadically used to supplement care, providing ongoing support, monitoring, or education. They noted that the integration of these tools varied widely across departments. A common concern shared by healthcare professionals was the insufficient funding for lifestyle interventions, which they felt limited the scope of what could be achieved.

“A lifestyle front office would make a huge difference. Patients need consistent advice, and we need the tools to provide it.” - Therapist

## Discussion

The aim of this study was to explore the experiences and preferences of healthcare professionals involved in the care of patients with cancer or osteoarthritis regarding^1^ the content of lifestyle interventions to support healthy behaviors during the perioperative period and^2^ the optimal organizational and social context for delivering these interventions. Healthcare professionals highlighted several interrelated challenges, including limited time, inadequate training in broader lifestyle factors, and the need for additional skills such as motivational interviewing. They expressed difficulties in initiating lifestyle discussions, particularly around sensitive topics, due to a lack of confidence and structured tools. Additionally, barriers such as fragmented collaboration, organizational constraints, and limited resources were cited as impediments to effective implementation. They identified socioeconomic status, physical and social conditions, and health literacy as determinants of patients’ capability to initiate and maintain lifestyle changes. While surgery often served as a motivator for short-term changes, sustaining this motivation postoperatively remained a significant challenge, underscoring the importance of ongoing professional and social support.

Healthcare professionals identified systemic barriers for implementing lifestyle interventions, including insufficient time, resource limitations, and lack of financial support. The lack of reimbursement for prehabilitation remains a challenge, highlighting the ongoing tension between recognizing the value of lifestyle interventions and securing resources for their implementation. As reported by healthcare professionals in this study, previous research has shown that professionals struggle to discuss lifestyle-related topics due to time constraints, limited training, and uncertainty about the effectiveness of interventions.^22^ These barriers are widely recognized, and evidence suggests that targeted training and institutional support can enhance professionals’ ability to integrate lifestyle discussions into routine care.^23,24,35^ Additionally, many professionals refer patients to one another for lifestyle advice, indicating a lack of clear role distribution in this area. Participants reported practical constraints in implementing lifestyle advice during preoperative care, including limited consultation time, unclear referral pathways, and organizational barriers. These barriers are consistent with previous findings, where time constraints, insufficient training, and uncertainty about effectiveness impede lifestyle counseling in clinical practice.^32,36^ These factors impede timely delivery of lifestyle interventions and highlight the need for streamlined workflows and clearer role delineation among healthcare professionals. This aligns with findings that emphasize the importance of clear role allocation and interprofessional collaboration to prevent fragmented care and improve patient outcomes.^37,38^ Maintaining lifestyle changes requires practical interventions, motivation, and social support. Guidance from a trained lifestyle professional can help patients stay engaged and make lasting behavioral changes.^23,24^ Additionally, international comparisons show that engagement in lifestyle counseling varies across healthcare systems, influenced by cultural and structural factors.^25^ Addressing these challenges require policy changes, including improved reimbursement, enhanced professional training, and institutional support. Integrating lifestyle interventions into routine care is essential for initiating and maintaining a healthy lifestyle in patients with cancer or osteoarthritis. Healthcare professionals highlighted difficulties in navigating referral pathways for lifestyle support, which contributes to fragmented care. Establishing a centralized lifestyle front office could provide a clear point of contact, improve coordination among providers, and facilitate timely access to appropriate interventions.

In this study, healthcare professionals identified patients’ socioeconomic status, physical and social conditions, and health literacy as key determinants of the capability of patients to initiate and maintain a healthy lifestyle. Low health literacy is widely recognized as a barrier to effective health management, affecting patient engagement, adherence, and outcomes.^39,40^ Patients with low health literacy often struggle to grasp medical information, resulting in reduced engagement, poor adherence to treatment, and worse outcomes.^41,42^ Previous research indicating that improving health literacy through educational programs and preoperative counseling can motivate patients to initiate healthier behaviors.^41^ Healthcare professionals emphasized that enhancing patient motivation is essential. They suggested interventions that explicitly link lifestyle improvements to recovery and quality of life. The context in which lifestyle interventions occur was another significant focus. Professionals consistently expressed that interventions should be patient-centered and delivered in settings that align with the patient’s daily life eventually by the appropriate disciplines in either secondary or primary care. They recommended community-based approaches, ensuring that care is provided in proximity to the patient’s environment. Research has shown that this approach improves adherence to treatment plans and enhances intrinsic motivation for making sustainable lifestyle changes,^43^ improving patient outcomes,^44^ and enhancing patient autonomy.^44,45^ Shared decision-making can personalize healthcare by giving patients the autonomy to make their own choices regarding their treatment.^46^

In the current study, healthcare professionals noted that surgery often serves as a short-term motivator for lifestyle change. Healthcare professionals acknowledged that while patients are often motivated to improve their health preoperatively, sustaining these changes postoperatively remains a significant challenge. This finding underscores the necessity of ongoing professional and social support to facilitate long-term adherence. Motivation is often higher before surgery, which supports beneficial lifestyle changes that improve surgical outcomes. However, healthcare professionals in this study observed that motivation tends to decrease after surgery, and postoperative support for long-term behavior change is limited. This highlights a gap in ongoing care and suggests the need for better follow-up to maintain healthy behaviors after surgery. Social support, particularly from loved ones and caregivers, was recognized as a critical factor in maintaining healthy behaviors. The importance of social relationships in the treatment of disease and the maintenance of a healthy lifestyle has drawn the attention of scientists and practitioners across a large number of behavioral science and health disciplines.^47,48^ Social support has been identified as an important contributor to general well-being that buffers the impact of stressful experiences, including those related to physical illness.^49^ While some professionals actively encourage peer support, others expressed concerns about the unregulated nature of online peer networks, which can sometimes disseminate misinformation. Sharing experiences is the essence of peer support and enables a peer to offer experiential empathy, something generally beyond the scope of health professionals.^50^ More structured integration of social, professional and peer support throughout the care process from preoperative to postoperative could help address this problem by improving information to professionals and patients. Although cancer and orthopedic healthcare professionals come from distinctly different specialties with varied treatment trajectories, our findings indicate largely shared perceptions regarding barriers and facilitators for lifestyle interventions. Future research could further explore specialty-specific needs to tailor interventions more precisely.

### Strengths and Limitations

This study provides qualitative insights into the experiences and preferences of healthcare professionals across multiple disciplines involved in pre- and postoperative care. The inclusion of diverse perspectives enables a comprehensive understanding of the challenges and opportunities in delivering lifestyle interventions during the perioperative period. However, several limitations should be considered. First, the study relied on self-reported data, which introduces the risk of social desirability bias, potentially overstating participants’ involvement or commitment to lifestyle discussions. This inherent limitation in qualitative self-reporting could affect the depth and authenticity of the insights gathered. Second, the geographic and institutional context may limit the generalizability of the findings, as healthcare systems and resources vary widely across regions. Another limitation was that while the study identifies gaps in interdisciplinary collaboration and referral pathways, it does not evaluate existing interventions, nor does it provide empirical evidence on the feasibility of proposed solutions like a centralized lifestyle front office. This highlights a need for future research to apply established frameworks from implementation science to systematically assess intervention feasibility, scalability, and impact. Finally, the absence of a formal risk of bias assessment tool reflects the exploratory nature of this qualitative research but should be considered when interpreting the findings. Future studies should incorporate such tools or mixed-methods approaches to enhance methodological rigor and address these limitations.

### Implications for Practice

Several practical and policy-oriented implications emerge from this study. Our findings highlight healthcare professionals’ recognition of patient health literacy as an important factor influencing engagement with lifestyle interventions. While this study did not assess the effectiveness of educational or counseling programs, enhancing health literacy may support patients in understanding and adopting healthy behaviors. Future research should further investigate the impact of such initiatives within perioperative care. Moreover, healthcare professionals require targeted training to address the unique needs of patients with limited health literacy. Healthcare professionals indicated a need for enhanced communication skills to facilitate lifestyle discussions. While motivational interviewing is one approach, evidence regarding its effectiveness in routine clinical training is mixed.^51^ Therefore, tailored communication training should consider context-specific needs and available resources. The implementation of lifestyle interventions should emphasize community-based care, leveraging platforms like to streamline referrals and improve access to appropriate specialists. Additionally, the lack of clear role distribution among healthcare professionals in providing lifestyle advice should be addressed. Establishing clearer responsibilities and interdisciplinary collaboration can prevent fragmentation of care and improve patient support. Finally, systemic changes are needed to address financial and organizational barriers to lifestyle interventions. Policymakers should consider integrating prehabilitation and other preventive programs into insurance coverage to ensure their scalability and sustainability. Addressing these challenges will require a concerted effort from healthcare professionals, institutions, and policymakers to create an environment where lifestyle interventions can be effectively implemented and maintained.

## Conclusion

This study highlights several challenges and opportunities in the integration of lifestyle interventions during the perioperative phase within healthcare for patients with cancer or osteoarthritis. Healthcare professionals emphasized the need for improved training, time management, and interprofessional collaboration to effectively discuss and implement lifestyle changes. Patient motivation, particularly in relation to surgery, emerged as a key factor, but maintaining long-term changes postoperatively remains difficult without sustained support. Socioeconomic factors, health literacy, and physical and social conditions were identified as significant barriers to patient capability in adopting healthy lifestyles. To overcome these challenges, the establishment of centralized lifestyle support systems, increased professional training, and patient-centered, community-based approaches are essential. Addressing these issues could lead to improved patient outcomes and long-term adherence to initiating and maintaining a healthy lifestyle.

## Data Availability

The data underlying this article will be shared on reasonable request to the corresponding author.*
